# Supplementary material for: Anodic Stripping Voltammetric Analysis of Trace Arsenic(III) on a Au-Stained Au Nanoparticles/Pyridine/Carboxylated Multiwalled Carbon Nanotubes/Glassy Carbon Electrode
Source: Nanomaterials (Basel). 2022 Apr 24;12(9):1450. doi: 10.3390/nano12091450 (PMC9105122; doi:10.3390/nano12091450)
Supplement: Supplementary file 1 [file nanomaterials-12-01450-s001.zip › nanomaterials-1656663-supplementary.pdf]

Supporting Information

Anodic Stripping Voltammetric Analysis of Trace Arsenic(III) on a Au-Stained Au Nanoparticles/Pyridine/Carboxylated Multiwalled Carbon Nanotubes/Glassy Carbon Electrode

Yun Du [1,2], Chenglong Sun [1], Yuru Shen [1], Luyao Liu [1], Mingjian Chen [1], Qingji Xie [1,*] and Hongbo Xiao [3,*]

[1] Key Laboratory of Chemical Biology & Traditional Chinese Medicine Research, Ministry of Education of China, College of Chemistry and Chemical Engineering, Hunan Normal University, Changsha 410081, China; hello_dudu1990@163.com (Y.D.); 15588654717@163.com (C.S.); shenyuru163@163.com (Y.S.); lly19970214@163.com (L.L.); mingjian412@163.com (M.C.)

[2] Changsha Center for Diseases Prevention and Control, Changsha 410004, China

[3] College of Science, Central South University of Forestry and Technology, Changsha 410004, China

* Correspondence: xieqj@hunnu.edu.cn (Q.X.), anabeel@126.com (H.X.)

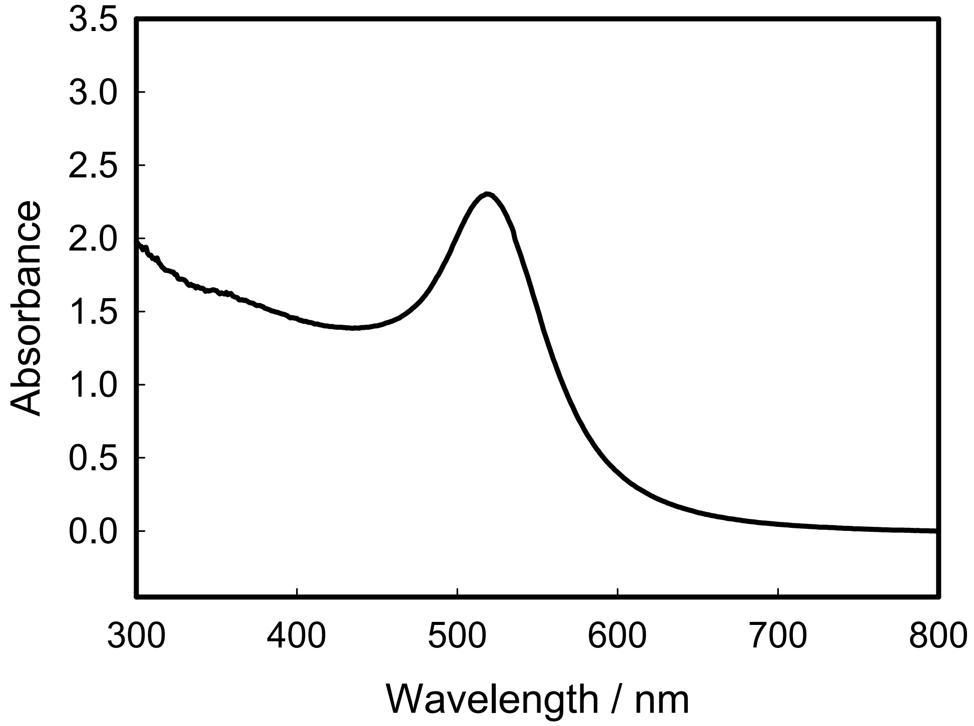

Figure S1. UV-Vis spectrum of the AuNPs.

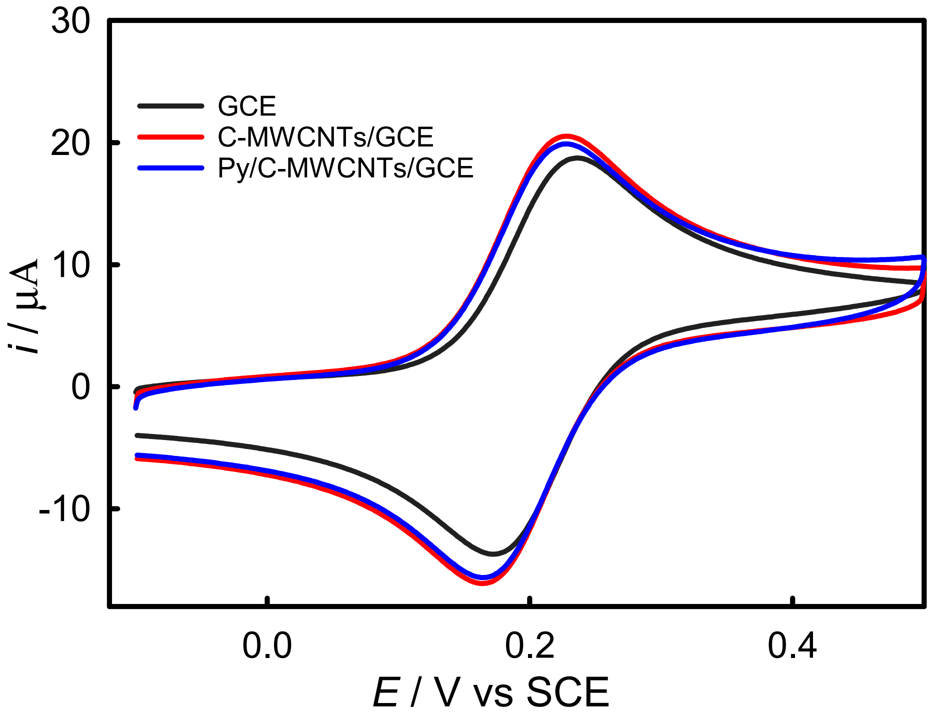

Figure S2. CV curves on GCE, C-MWCNTs/GCE and Py/C-MWCNTs/GCE in 0.1 M PBS containing 1.0 mM $K_4Fe(CN)_6$ at pH 7.0.

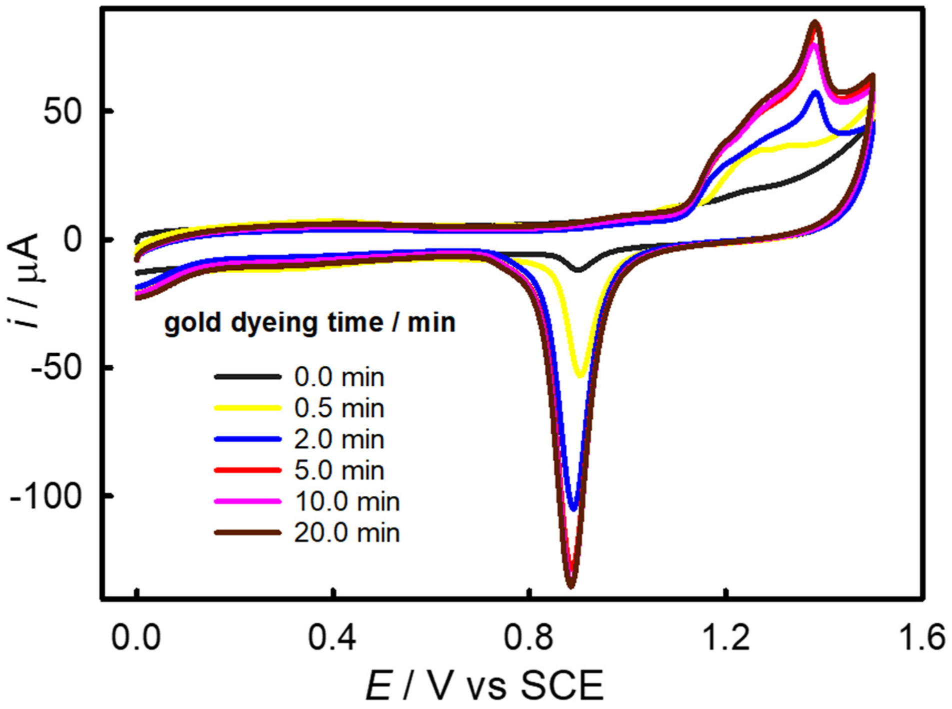

Figure S3. CV curves on Au_s/Py/C-MWCNTs/GCE prepared at different gold staining time. Scan rate: 50 mV s^{-1}.

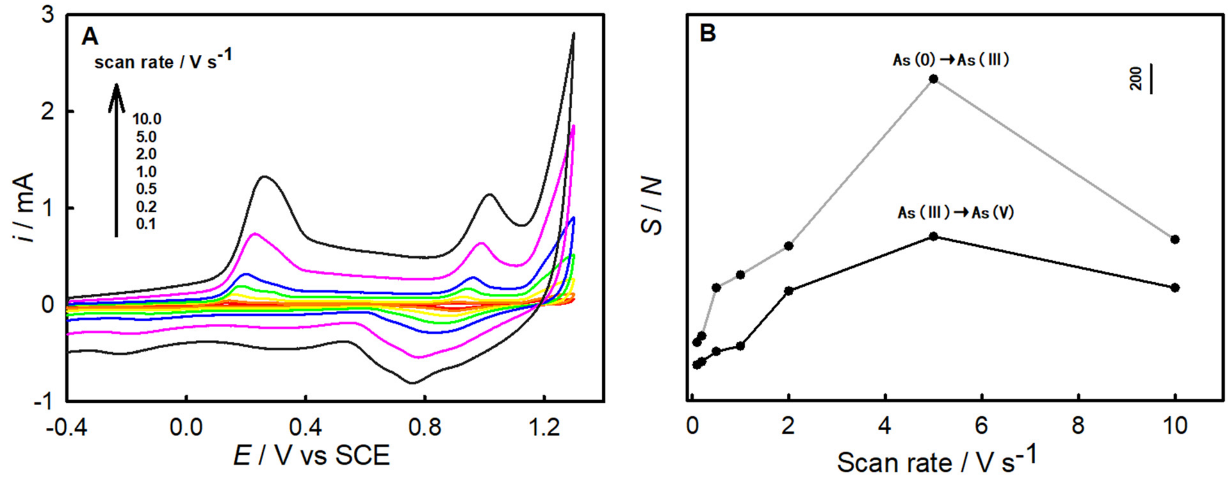

Figure S4. (**A**) CV curves on Au$_s$/Py/C-MWCNTs/GCE in 0.1 M aqueous H_2SO_4 containing 1.0 μM As(III) at different scan rates, (**B**) the associated *S*/*N* plots of As(0)→As(III) (grey) and As(III)→As(V) (black) *vs* scan rate, *S*: peak current, *N:* noise of the background current. The experiments were conducted after preconcentration at −0.40 V for 7 min.

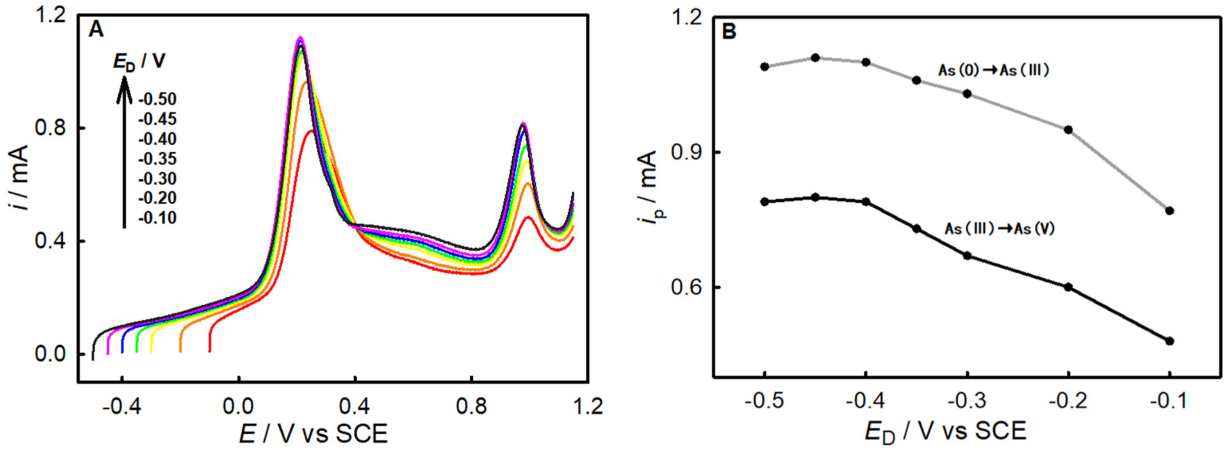

Figure S5. LSASV responses (**A**) on Au_s/Py/C-MWCNTs/GCE in 0.1 M aqueous H_2SO_4 containing 1.0 μM As(III) at various As(0)-deposition potential (E_D) values and the corresponding peak currents versus E_D (**B**). As(0)-deposition time (t_D) = 7 min.

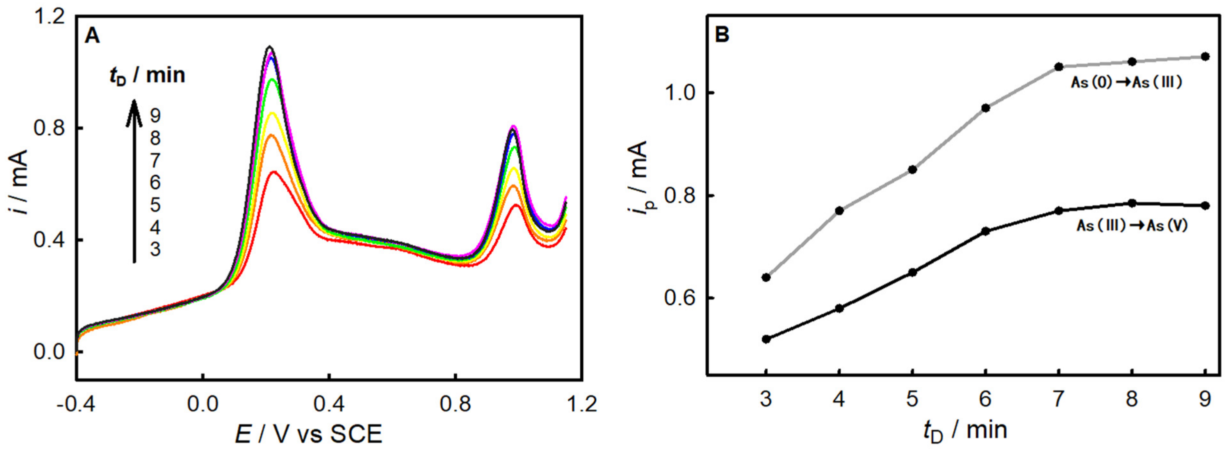

Figure S6. LSASV responses (**A**) on Au_s/Py/C-MWCNTs/GCE in 0.1 M aqueous H_2SO_4 containing 1.0 μM As(III) for various As(0)-deposition time (t_D) and the corresponding peak currents versus t_D (**B**). $E_D = -0.40$ V.

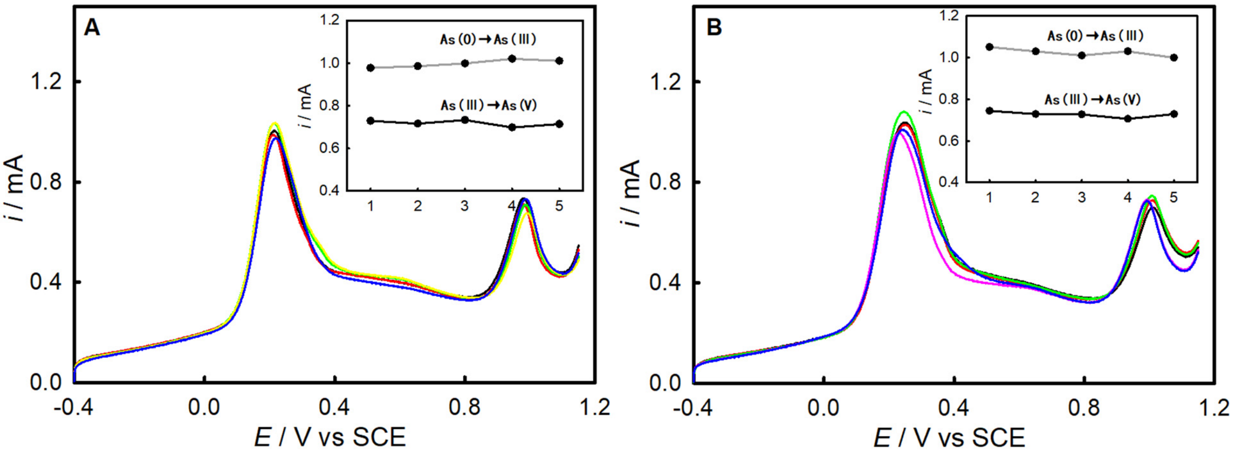

Figure S7. Stability and reproducibility of LSASV response of 1.0 μM As(III). (**A**) Repeated five times on one Au_s/Py/C-MWCNTs/GCE in 0.1 M aqueous H_2SO_4, (**B**) On a batch of five different Au_s/Py/C-MWCNTs/GCEs. E_D = −0.40 V, t_D = 7 min.

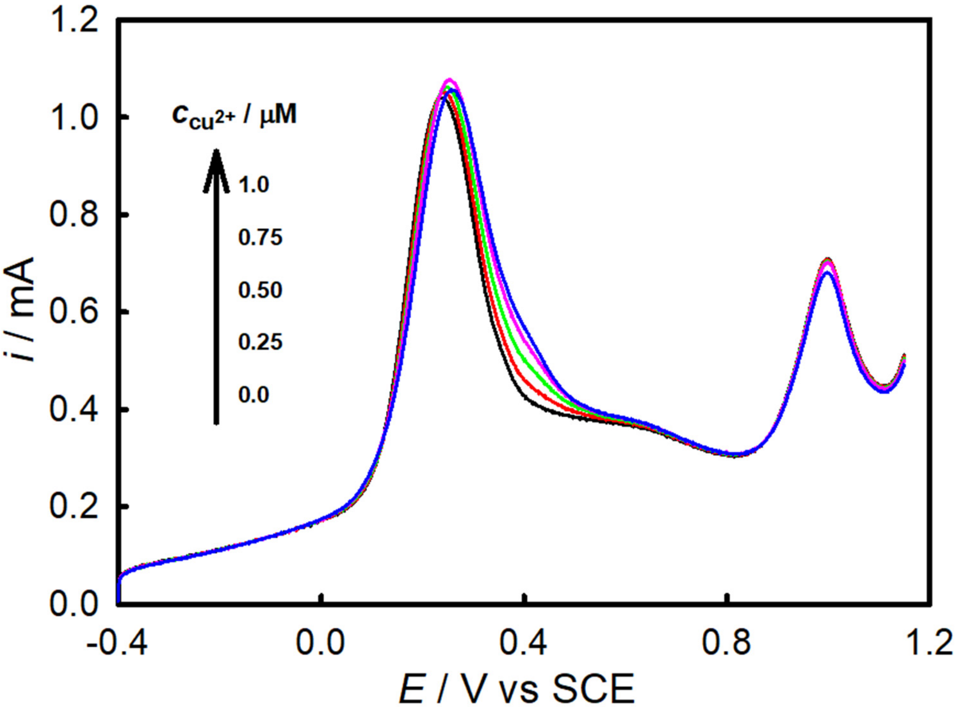

Figure S8. LSASV response on Au_s/Py/C-MWCNTs/GCE in 0.1 M aqueous H_2SO_4 containing 1.0 μM As(III) with an interval addition of 0.25 μM Cu^{2+}. E_D = −0.40 V, t_D = 7 min.
